# A molecular and ecological study of *Macracanthorhynchus ingens* (von Linstow, 1879) (Acanthocephala: Archiacanthocephala), in its paratenic and definitive hosts in southeastern Mexico and the Eastern USA

**DOI:** 10.1007/s11230-023-10104-5

**Published:** 2023-06-20

**Authors:** Mirza Patricia Ortega-Olivares, Yanet Velázquez-Urrieta, Ana Lucia Sereno-Uribe, Michael B. Harvey, Martín García-Varela

**Affiliations:** 1 Departamento de Zoología, Instituto de Biología, Universidad Nacional Autónoma de México, Ciudad Universitaria, C. P. 04510, Mexico City, Mexico; 2 Department of Biological Sciences, Broward College, 3501 S.W. Davie Road, Davie, FL USA

## Abstract

The acanthocephalan *Macracanthorhynchus ingens* (von Linstow 1879) (Acanthocephala: Archiacanthocephala) is a parasite that infects the gut of carnivores (racoons, coyotes, wolves, foxes, badgers, skunks, opossum, mink and bears) as an adult and the body cavity of lizards, snakes, and frogs as a cystacanth in the Americas. In this study, adults and cystacanths of *M. ingens* from southeastern Mexico and southern Florida, USA, were identified morphologically by having a cylindrical proboscis armed with 6 rows of hooks each with 6 hooks. Hologenophores were used to sequence the small (SSU) and large (LSU) subunits of ribosomal DNA and cytochrome c oxidase subunit 1 (*cox 1*) from mitochondrial DNA. Phylogenetic analysis of the new SSU and LSU sequences of *M. ingens* placed them in a clade with other sequences available in GenBank identified as *M. ingens*. The *cox 1* tree showed that the nine new sequences and six previously published sequences of *M. ingens* from the USA form a clade with other sequences previously identified as *M. ingens* from GenBank*.* The intraspecific genetic divergence among isolates from the Americas ranged from 0 to 2%, and in combination with the phylogenetic trees confirmed that the isolates belonged to the same species. The *cox 1* haplotype network inferred with 15 sequences revealed 10 haplotypes separated from each other by a few substitutions. Rio Grande Leopard Frogs and Vaillant´s Frogs harbored cystacanths with low prevalence, 28% and 37% respectively, in Mexico. Brown Basilisks, an invasive lizard in Florida, USA, had high values of prevalence, 92% and 93% in males and females, respectively. Females harbored more cystacanths than males (0–39 *vs* 0–21) for unknown reasons that may, however, be related to ecological differences.

## Introduction

Acanthocephalans of the class Archiacanthocephala Meyer 1931 are among the most common parasites of terrestrial birds and mammals. Within archiacanthocephalans, *Macracanthorhynchus* Travassos 1917 is a large, globally distributed genus and common parasite in the intestine of mammals. At present, the genus includes four species: *M. hirudinaceus* (Pallas 1781) Travassos 1917 (type species), *M. catulinus* Kostylew 1927, *M. rinaceid* Dollfus 1953 and *M. ingens* (von Linstow 1879) Meyer 1932 (Amin, 2013). Species of *Macracanthorhynchus* have been the target of numerous studies related to their ecology, host-parasite relationships, pathogenicity, taxonomy, and systematics (Schmidt, 1971; Near et al., 1998; Richardson, 2005, 2014; García-Varela & Nadler, 2005; Kennedy, 2006; Migliore et al., 2021; Dessì et al., 2021). *Macracanthorhynchus ingens* was described from the intestine of a racoon (*Procyon lotor* Linnaeus) from North America (see Richardson, 2014), and since then this acanthocephalan has been recorded in other definitive hosts such as black bear (*Ursus americanus* Pallas), domestic dog (*Canis familiaris* Linnaeus), coyote (*Canis latrans* Say), hog-nosed skunk (*Conepatus leuconotus* Lichtenstein), eastern striped skunk (*Mephitis mephitis* Schreber), mink (*Neovison vison* Schreber), hairy-tailed mole (*Parascalops breweri* Bachman), spotted skunk (*Spilogale gracilis* Merriam) and gray fox (*Urocyon cinereoargenteus* Schreber) in diverse countries such as Canada, United States, Mexico, Nicaragua and Colombia (García-Varela & Nadler, 2005; Richardson, 2014; Richardson et al., 2017; Hartnett et al., 2018). The life cycle of *M. ingens* is well known, adult worms live and reproduce sexually in the digestive tract of their definitive host. Mature eggs are expelled into the environment with the feces of the definitive host. After the ingestion of the eggs by an arthropod (millipedes, beetles, wood roaches) intermediate host, the parasite develops into a cystacanth (larval form). The intermediate host is ingested by a paratenic host (lizards, snakes, and frogs). Finally, the paratenic host is eaten by the definitive host and the life cycle is completed (Moore, 1946; Richardson, 2005, 2014; Richardson et al., 2016).

During a survey of parasitic helminths in southeastern Mexico, three adult specimens of an acanthocephalan were recovered from the digestive tract of two common raccoons (*Procyon lotor* Linnaeus) and cystacanths from the body cavities of Vaillant´s Frog (*Lithobates vaillanti* Brocchi) and Rio Grande Leopard Frog (*Lithobates berlandieri* Baird) with low values of prevalence. In addition, other cystacanths were recovered from the body cavity of the Brown Basilisk (*Basiliscus vittatus* Wiegmann), in Southern Florida, United States. After a morphological examination of worms from both stages, adults and cystacanths were identified as *Macracanthorhynchus ingens*. Therefore, the objectives of this study were: *i*) to compare morphologically the cystacanths recovered from the frogs and lizard from the southeastern Mexico and from southern Florida, United States; *ii*) link the cystacanths with adults recovered from the raccoons in southeastern Mexico; *iii*) test the systematic position of *M. ingens* within Archiacanthocephala by using small (SSU) and large (LSU) subunit from nuclear ribosomal DNA; *iv*) explore the genetic structure of the populations by using sequences of the cytochrome c oxidase subunit 1 (*cox1*) from mitochondrial DNA; and *v*) discuss the ecological parameters of the infection of *M. ingens* in its paratenic hosts.

## Materials and methods

### Sample collection

During several field expeditions in 2018, 2019 and 2020, two common raccoons (*Procyon lotor*) (18° 15′ 23.7″ N, 96° 23′ 33″ W), seven adult male Rio Grande Leopard Frogs (*Lithobates berlandieri*), and eight adults female Vaillant´s Frogs (*Lithobates vaillanti*) were collected in southeastern Mexico (18º 35′ –18 º 36′ N, 95 º 05′ –95º 06′ W). In March, October, and November of 2021 and October and November of 2022, 33 Brown Basilisk (*Basiliscus vittatus*) were collected from two ponds in Davie, Florida, U.S.A. Broward County: Davie, pond at northeast corner of junction of Hiatus road and SW 36th Street (26° 07′ 60.2″ N, 80° 29′ 77″ W) and Broward College, Central Campus, ponds on N end of campus (26° 08′ 41″ N, 80° 23′ 38″ W). The sample of basilisks included seven juveniles (snout-vent length [SVL] 67–90, 74 ± 9 mm), 12 adult males (SVL 107–142, 127 ± 11 mm), and 14 adult females (SVL 110–139, 118 ± 8 mm). The definitive and paratenic hosts were dissected. Their viscera were placed in separate Petri dishes with a 0.75% saline solution and examined under a dissecting microscope. The acanthocephalans were removed from the intestine (adult stage) and from the body cavity (encysted cystacanths) and washed in a 0.75% saline solution. Later, the unencysted cystacanths were placed in distilled water at 4°C overnight and subsequently were fixed and preserved in 70 or 100% ethanol.

### Morphological analyses

A few acanthocephalans were gently punctured with a fine needle, stained with Mayer’s paracarmine, destained in 70% acid ethanol, dehydrated in a graded ethanol series, cleared in methyl salicylate and mounted on permanent slides with Canada balsam. Each slide with a cystacanth was deposited in the Colección Nacional de Helmintos, Instituto de Biología, Universidad Nacional Autónoma de México, Mexico City, under numbers (CNHE, 11888–11895). Additional samples of unstained cystacanths were deposited at the Museum Southwestern Biology, Parasite Collection (under number MSB, 35989–35990). In addition, vouchers of our sample of Brown Basilisks were deposited in the Museum Southwestern Biology, USA, under numbers (MSB, 35979–35988, 35991–35997).

The cystacanths were analysed with a Leica DM 1000 LED microscope equipped with bright field (Leica, Wetzlar, Germany). The acanthocephalans were identified by conventional morphological criteria following the study of Moore (1946). For scanning electron microscopy (SEM), two cystacanths were individually dehydrated with an ethanol series, critical point dried, sputter coated with gold, and examined with a Hitachi Stereoscan Model S-2469N scanning electron microscope operating at 15 kV at the Instituto de Biología, Universidad Nacional Autónoma de México (UNAM).

### DNA sequence generation

A total of nine specimens identified as *M. ingens* were analyzed. Before DNA extraction, a tissue fragment was cut from three cystacanths and two adults from southeastern Mexico and two cystacanths from southern Florida, USA (hologenophores, Pleijel et al., 2008), whereas the rest of the body was stained with Mayer’s paracarmine and mounted on permanent slides with Canada balsam. Two other specimens identified as *M. ingens* from southern Florida, USA, and two specimens identified as *Oncicola* sp., from southeastern Mexico were placed individually in tubes and digested overnight at 56 °C in a solution containing 20 mM NaCl, 100 mM Na_2_ EDTA (pH 8.0), 10 mM Tris–HCl (pH 7.6), 1% sarkosyl, and 0.1 mg/ml proteinase K. Following digestion, genomic DNA was extracted from the supernatant using the DNAzol reagent (Molecular Research Center, Cincinnati, OH, USA) according to the manufacturer’s instructions. Two regions of nuclear ribosomal DNA (rDNA) and one mitochondrial DNA region (mtDNA) were amplified using the polymerase chain reaction (PCR). A near complete fragment from the small subunit from 18S rDNA (~1,800 bp; SSU) was amplified using two overlapping PCR fragments of 1,000 bp: the SSU amplicon 1 using the forward primer 5′-AGA TTA AGC CAT GCA TGC GT-3′ and reverse primer 5′-AAC TTT TCG TTC TTG ATT AA TG-3′ and, the SSU amplicon 2 using the forward primer 5′-GCA GCG CGG TAA TTC CAG CTC-3′ and reverse primer 5′-GCA GGT TCA CCT ACG GA AA-3′ (García-Varela & Nadler, 2005). A near complete fragment of the large subunit from 28S rDNA (~2,900 bp; LSU) was amplified using three overlapping PCR fragments of 1200-1300 bp: the LSU amplicon 1 using the forward primer 5′-CAA GTA CCG TGA GGG AAA GTT GC-3′ and reverse primer 5′-CAG CTA TCC TGA GGG AA AC-3′, the LSU amplicon 2 using the forward primer 5′-ACC CGA AAG ATG GTG AAC TA TG-3′ and the reverse primer 5′- CTT CTC CAA CGT CAG TCT TC AA-3′, and, the LSU amplicon 3 using the forward primer 5′- CTA AGG AGT GTG TAA CAA CTC ACC-3′ and reverse primer 5′-CTT CGC AAT GAT AGG AAG AG CC-3′ (García-Varela & Nadler, 2005). Finally, the cytochrome c oxidase subunit 1 (*cox 1*) from the mitochondrial DNA was amplified using the forward primer 5′-AGTTCTAATCATAA(R)GATAT(Y)GG-3′ and reverse primer 5′ -TAAACTTCAGGGTGACCAAAAAATCA-3′ (Folmer et al., 1994). PCR amplifications were performed in a total volume of 25 μl containing 2 μl of each primer, 10 pmol/ μl, 2.5 µl of 10X buffer, 1.5 μl of 2 mM MgCl_2_, 2 μl of the genomic DNA and 1U of Taq DNA polymerase (Platinum Taq, Invitrogen Corporation, California, United States). PCR cycling parameters for rDNA amplifications included denaturation at 94 °C for 3 min, followed by 35 cycles of 94 °C for 1 min, annealing at 50–58 °C (optimized for each fragment amplified) for 1 min, and extension at 72 °C for 1 min, followed by a post-amplification incubation at 72 °C for 7 min. Sequencing reactions were performed with the primers mentioned above using ABI Big Dye (Applied Biosystems, Boston, Massachusetts) terminator sequencing chemistry. Reaction products were separated and detected using an ABI 3730 capillary DNA sequencer. Contigs were assembled and base-calling differences resolved using Codoncode Aligner version 9.0.1 (Codoncode Corporation, Dedham, Massachusetts).

### Alignments, phylogenetic analyses, haplotype network and ecological analyses

Newly generated sequences of SSU, LSU and *cox 1* were aligned with published sequences for other members of Archiacanthocephala retrieved from the GenBank dataset (Table 1). Alignments for each molecular marker (SSU, LSU and *cox 1*) were constructed using the software Clustal W (Thompson et al., 1994). A nucleotide substitution model was selected for the dataset using jModelTest version 2.1.7 (Posada, 2008). Phylogenetic analyses were inferred through maximum likelihood (ML) with the program RAxML version 7.0.4 (Stamatakis, 2006). A GTRGAMMAI substitution model was used, and 10,000 bootstrap replicates were run to assess nodal support. In addition, a Bayesian analysis was carried out, using the program MrBayes 3.2.2 (Ronquist et al., 2012) with two Markov chain Monte Carlo (MCMC) runs for 10 million generations, sampling every 1000 generations, a heating parameter value of 0.2 and a burn-in of 25%. The resulting phylogenetics trees were visualized and edited using FigTree version 1.4.2 (Rambaut & Drummond, 2007). Finally, uncorrected *p* distances were estimated using the MEGA program (Kumar et al., 2016). To explore whether paratenic hosts from both the Mexican and Floridian localities share the same *cox*1 haplotypes, an unrooted statistical network was constructed using PopART (Leigh & Bryant, 2015) with the minimum spanning network option (Bandelt et al., 1999).

**Table 1:** Classification and GenBank accession numbers of the specimens used in the phylogenetic analysis and haplotype network

Class	Order	Species	SSU	LSU	*cox 1*	References
**Rotifera**						
Monogononta	Ploima	*Asplanchna sieboldi*	AF092434	–	–	García-Varela et al. (2000)
			–	AY829085	–	García-Varela & Nadler (2005)
			–	–	AF416994	García-Varela (unpublished data)
		*Brachionus patulus*	AF154568	–	–	García-Varela et al. (2000)
			–	AY829084	AF416995	García-Varela & Nadler (2005)
		*Brachionus plicatilis*	AY218118	–	–	Giribet et al. (2004)
		*Lecane bulla*	–	AY829083	–	García-Varela & Nadler (2005)
Pararotatoria	Seisonacea	*Seison nebaliae*	DQ089737	DQ089744	DQ089730	García-Varela & Nadler (2006)
**Acanthocephala**						
Archiacanthocephala	Gigantorhynchida	*Mediorhynchus africanus*	KC261353	–	–	Amin et al. (2013)
		*Mediorhynchus gallinarum*	KC261354	–	KC261352	Amin et al. (2013)
		*Mediorhynchus* sp.	AF064816	–	AF416996	García-Varela et al. (2000)
			–	AY829087	–	García-Varela & Nadler (2005)
	Moniliformida	*Mediorhynchus grandis*	AF001843	–	–	Near et al. (1998)
		*Gigantorhynchus echinodiscus*	–	MK635344	–	Gomes et al. (2019)
		*Moniliformis kalahariensis*	–	–	MH401040	Amin et al. (2019)
		*Moniliformis moniliformis*	HQ536017	–	–	Foronda (unpublished data)
			Z19562	–	–	Telford & Holland (1993)
			–	AY829086	–	García-Varela & Nadler (2005)
			–	–	AF416998	García-Varela (unpublished data)
		*Moniliformis necromysi*		MT808220	MT803593	Gomes et al. (2020)
		*Moniliformis saudi*	KU206782	–	KU206783	Amin et al. (2016)
		*Moniliformis cryptosaudi*	MH401043			Amin et al. (2019)
		*Moniliformis ibunami*	MW136271 MW136272	MW136276 MW136277	MW115575 MW115576	Lynggaard et al. (2021)
	Oligacanthorhynchida	*Macracanthorhynchus hirudinaceus*	LC350002	LC350002	LC350002 MZ683370-75	Kamimura et al. (2018) Mehmood and Varcasia (unpublished data)
		*Macracanthorhynchus ingens*	AF001844	–	–	Near et al. (1998)
			–	AY829088	–	(García-Varela & Nadler (2005)
			– –	– –	AF416997 ON197103	García-Varela (unpublished data ) Najjari & Solgi (unpublished data)
			–	–	KT881246-49	Richardson et al. (2016)
			**OR077291**	**OR077292**	**OR077684** **OR077685** **OR077686** **OR077687** **OR077688** **OR077689** **OR077690** **OR077691** **OR077692**	**This study**
		*Oligacanthorhynchus microcephala*	AF064817	–	– KT881245	García-Varela et al. (2000) Richardson et al. (2016)
			–	AY829090	–	García-Varela & Nadler (2005)
					AF416998	
		*Oncicola* sp.	AF064818	–	–	García-Varela et al. (2000)
			–	AY829089	–	García-Varela & Nadler (2005)
			– –	– –	AF417000 **OR077693** **OR077694**	García-Varela (unpublished data) **This study**
		*Prosthenorchis elegans*	–	–	KT818504	(Falla et al. (2015)
		*Prosthenorchis* sp.	–	–	KP997253	Sokolov et al. (unpublished data)

Sequences in bold were generated in this study

The samples from the Brown Basilisks harbored a great number of cystacanths, allowing further investigation into this parasite’s population structure. For these analyses, we used the PAST statistical software package (Hammer et al., 2001). We used Welch’s *t*-test to compare counts of cystacanths between males and females. To determine if count of cystacanths increases with size of the paratenic host, we attempted linear regression analysis. Prior to this analysis, we tested assumptions of linearity (using the Durbin-Watson test), homoscedasticity (using the Breusch-Pagan test), and normality of residuals (using the Shapiro-Wilk test).

## Results

### Morphological identification

**Fig. 1:**
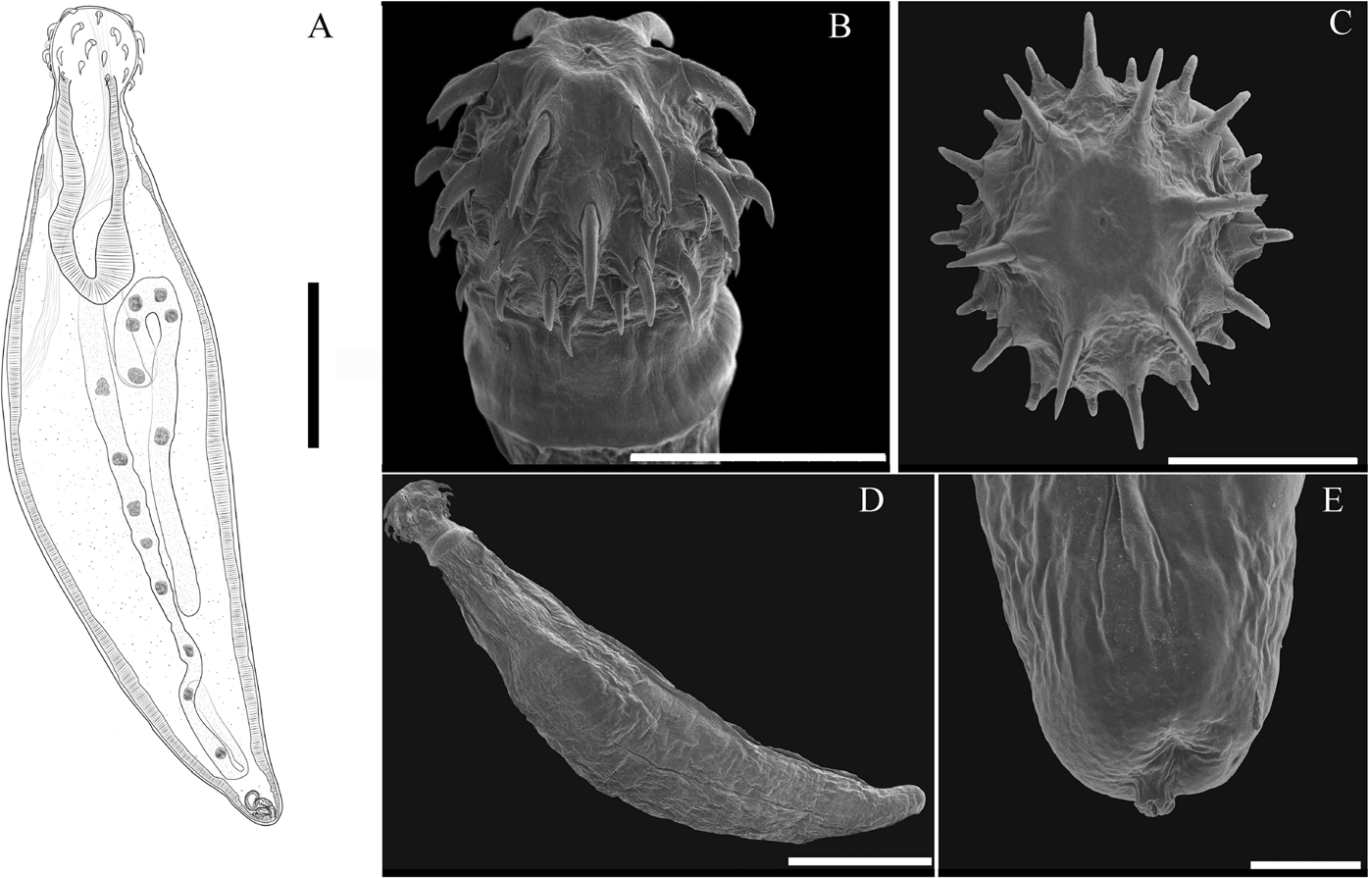
Drawing of *Macracanthorhynchus ingens* from *Basiliscus vittatus*. Cystacanth, total view (**A**); Scanning electron micrographs of the proboscis of a cystacanth of *Macracanthorhynchus ingens* (**B, C**); cystacanth total view (**D**); cystacanth posterior end (**E**). Scale bars = 1.0 mm (A); 400 µm (B); 300 µm (C); 1.0 mm (D); 100 µm (E).

Two carcasses of common raccoon found on the freeway were collected with a poor state of preservation. Two male and one female acanthocephalans were recovered from their intestines. These adults had partial or completely invaginated proboscids. In contrast, cystacanths from the frogs and lizard were alive when collected. The cystacanths showed similar morphological characteristics compared with those assigned to *M. ingens* by Richardson (2005), including (*i*) an elongated cylindrical trunk with a narrow posterior region; (*ii*) proboscis cylindrical; (*iii*) double-walled proboscis receptacle; (*iv*) hooks arranged in 6 rows, with 6 hooks per row; and (*v*) lemnisci very long extending to the posterior region, with small nuclei (Figs. 1A-D). Compared to previous descriptions, our specimens exhibited variability in body size, proboscis and hooks size (Table 2).

**Table 2:** Comparative measurements of specimens of *Macracanthorhynchus ingens*, *n* = number of specimens used. All measurements are in micrometers

Reference characters	Richardson, 2005 Adult	Richardson, 2005 Cystacanths	This study Cystacanths, Florida, USA *n* = 7	This study Cystacanths, Mexico *n* = 3
Body Length			4097–4952 (4522)	4057–4776 (4416)
Body Width			1350–1506 (1435)	1443–2789 (2116)
Proboscis Length	405–459 (437)	390–546 (467)	643–765 (733)	541–742 (648)
Proboscis Width	653–729 (683)	504–700 (590)	510–615 (557)	579–742 (670)
Proboscis receptacle Length			967–1234 (1075)	1141–1338 (1272)
Proboscis receptacle Width			438–594 (501)	378–510 (446)
Proboscis hooks size				
Row 1	160–212 (185)	153–212 (182)	110–117 (113)	175–208 (193)
Row 2	149–207 (182)	151–196 (173)	133–152 (143)	158–165 (162)
Row 3	104–158 (135)	117–158 (137)	110–119 (114)	118–165 (138)
Row 4	108–158 (123)	95–133 (114)	98–109 (104)	88–130 (99)
Row 5	86–106 (96)	86–104 (95)	89–94 (90)	63–69 (67)
Row 6	72–99 (86)	59–90 (82)	63–78 (67)	50–58 (55)

### Phylogenetic analyses and haplotype network

**Fig. 2:**
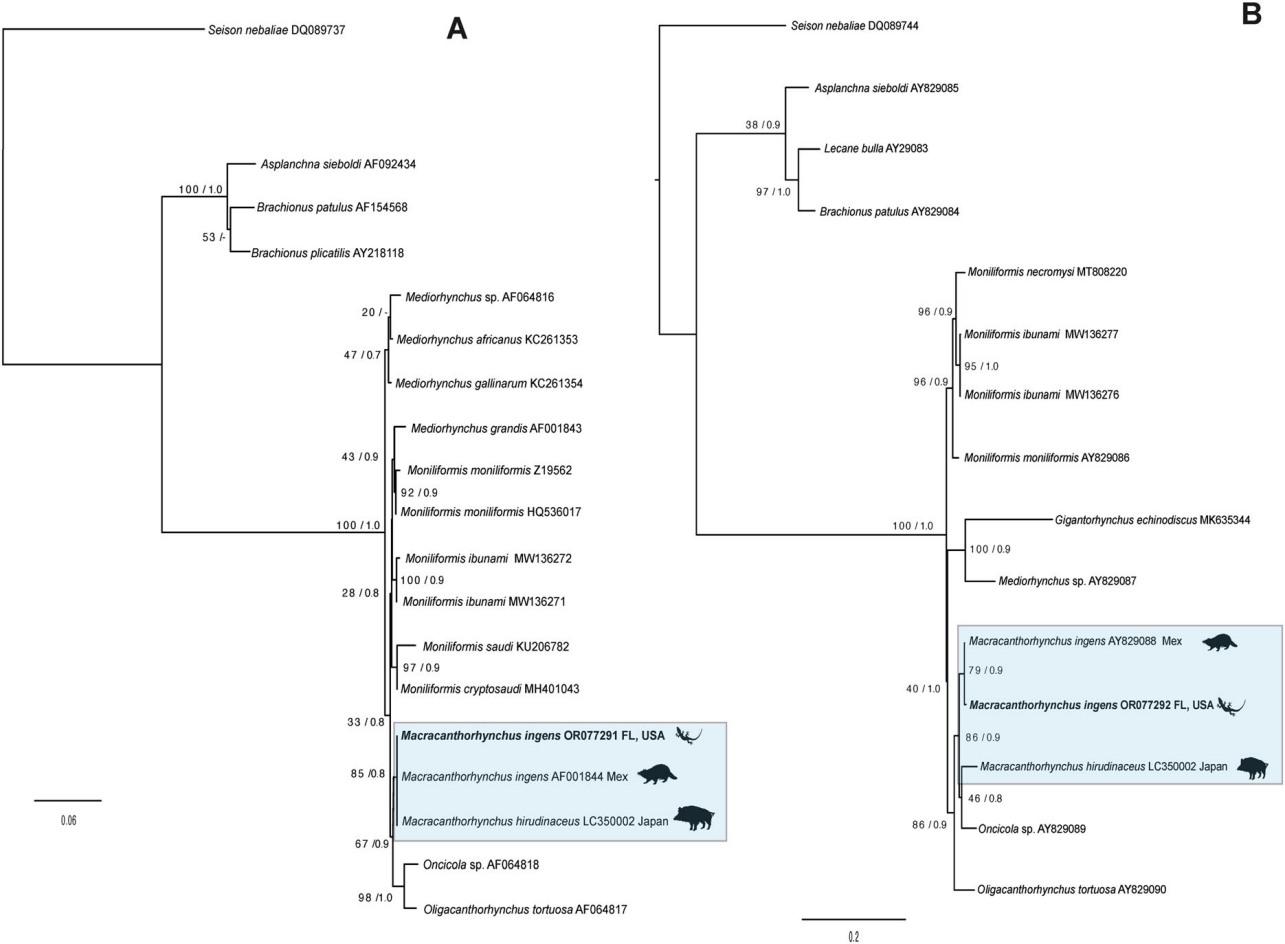
Phylogenetic trees using maximum likelihood (ML) and consensus Bayesian. SSU dataset (**A**), and LSU dataset (**B**). Numbers near internal nodes show ML bootstrap percentages/ Bayesian posterior probabilities.

The newly generated sequence from SSU was analysed together with 18 published sequences from 16 species, forming an alignment of 1,842 sites. The best evolution model was TIM +I+G. This data set included genera representing three orders of Archiacanthocephala, i.e., Moniliformida Schmidt 1972 (*Moniliformis* Travassos 1915), Gigantorhynchida Southwell et Macfine 1925 (*Mediorhynchus* Van Cleave 1925), and Oligacanthorhynchida Petrochenko 1956 (*Oligacanthorhynchus* Travassos 1915, *Oncicola* Travassos 1916, and *Macracanthorhynchus*) (Table 1). The phylogenetic trees inferred with the SSU showed that three genera from Oligacanthorhynchida are monophyletic. The new SSU sequences of *M. ingens* from Florida formed a clade together with another isolate identified as *M. ingens* (GenBank:AF001844) recovered from a common raccoon in southeastern Mexico by García-Varela et al. (2000) and an isolate of *M. hirudinaceus* (GenBank: LC350002) recovered from the Japanese boar (*Sus scrofa leucomystax* Temminck) from Japan. However, this clade was unresolved due these three isolates sharing the same node. This clade is sister to a clade formed by two sequences of the genera *Oligacanthorhynchus* and *Oncicola* from Oligacanthorhynchida (Fig. 2A).

The newly generated sequence from LSU was analysed together with 14 published sequences representing 14 species, forming an alignment of 2,894 sites. The best evolution model was GTR+G+I. The topologies inferred with the LSU data set yielded substantial differences relative to the topologies inferred with the SSU data set, which could be due to the number of taxa sampled or that some sequences are partial. Unlike the SSU tree, *Macracanthorhynchus* is paraphyletic in the LSU tree with *M. hirudinaceus* placed in a weakly supported clade with *Oncicola* sp*.* Both the LSU and SSU trees supported the monophyly of Oligacanthorhynchida. The Mexican and Florida samples of *M. ingens* were recovered as sister lineages in the LSU tree. (Fig. 2B).

**Fig. 3:**
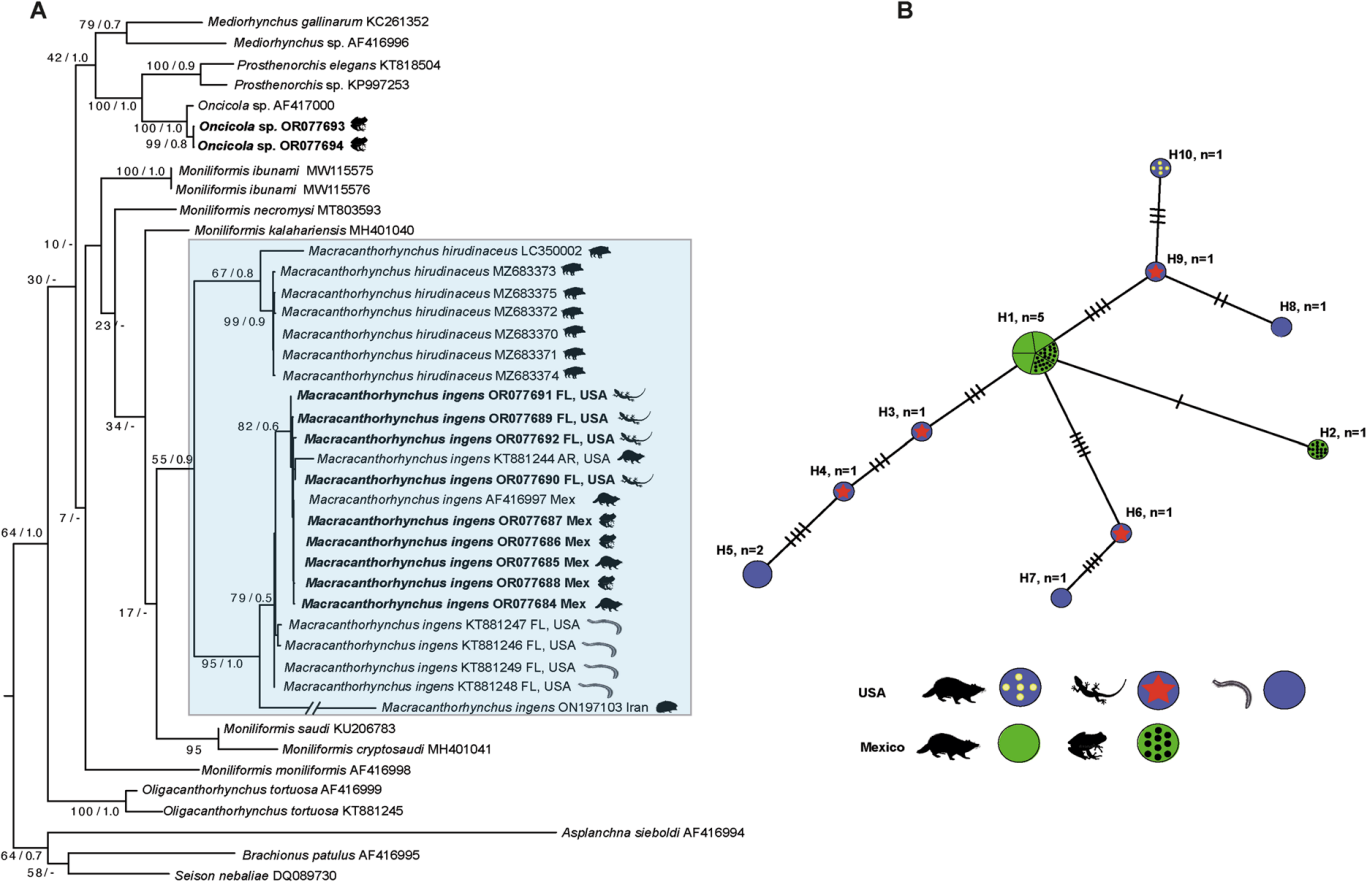
Phylogenetic trees using maximum likelihood (ML) and consensus Bayesian Inference for the *cox* 1 dataset (**A**). Numbers near internal nodes show ML bootstrap percentage values/ Bayesian posterior probabilities. Median-joining network of samples of *Macracanthorhynchus ingens* built with the *cox* 1 gene (**B**). Each circle represents a haplotype, with size proportional to the haplotype’s frequency in the populations.

The *cox 1* dataset included 664 sites and 42 sequences, and the best model was GTR + G + I. The tree inferred from the *cox 1* data set showed that Oligacanthorhynchida is paraphyletic, because *Oligacanthorhynchus*, *Oncicola*, and *Macracanthorhynchus* are nested in three independent clades (Fig. 3A). Our phylogenetic trees showed that the genus *Macracanthorhynchus* is monophyletic contains two main subclades. The first subclade is formed by seven isolates identified as *M. hirudinaceus* downloaded from GenBank (MZ683370-75) from wild boar (*Sus scrofa meridionalis* Forsyth Major) from Italy plus an isolate identified as *M. hirudinaceus* (LC350002) from a Japanese wild boar (*Sus scrofa leucomystax*) from Japan. The second subclade was formed by 16 *cox 1* sequences from specimens that we identified as *M. ingens* from southeastern Mexico and Florida, USA, as well as those available in the GenBank database identified as *M. ingens* from the USA (KT881244; KT881246-49) and Iran (ON197103) (Fig. 3A).

The uncorrected genetic divergence estimated with the *cox 1* data set between *M. hirudinaceus*, and *M. ingens* its sister taxa in the phylogenetic trees, ranged from 24 to 26%. The genetic divergence among our specimens of *M. ingens* recovered from two frog species and two racoons from southeastern Mexico ranged from 0 to 0.03%; among the isolates recovered from a raccoon, the basilisks, and Florida Ivory Millipede (*Chicobolus spinigerus* Wood) ranged from 0 to 2%. In contrast, the genetic divergence between an isolate identified as *M. ingens* (GenBank: ON197103) from a hedgehog from Iran, and those isolates from the Americas range from 8 to 20%. Based on monophyly and low genetic distances, the various sequences of *M. ingens* from North America almost certainly represent a single species.

The haplotype network built in this study was inferred with 15 specimens and 619 characters. (The sequence of *M. ingens* (GenBank: ON197103) from Iran was removed from the analysis because it contains only 248 bp). The network inferred herein recognized 10 haplotypes. The haplotypes were separated from each other by a maximum of five substitutions. The haplotypes H1 and H2 were found in southeastern Mexico. The most frequent haplotype (H1, n = 5) was found in three adult specimens and two cystacanths from southeastern Mexico. The Haplotypes H3–H10 were found in the United States. The H10 correspond to adult worms recovered from a raccoon in Arkansas. The H3, H4, H6 and H9 correspond to cystacanths recovered from basilisks (*Basiliscus vittatus*) in Florida. Finally, the H5, H7 and H8 were found in the intermediate host, the Florida ivory millipedes (*Chicobolus spinigerus*) (Fig. 3B).

### Ecological parameters of the infection with M. ingens

**Fig. 4:**
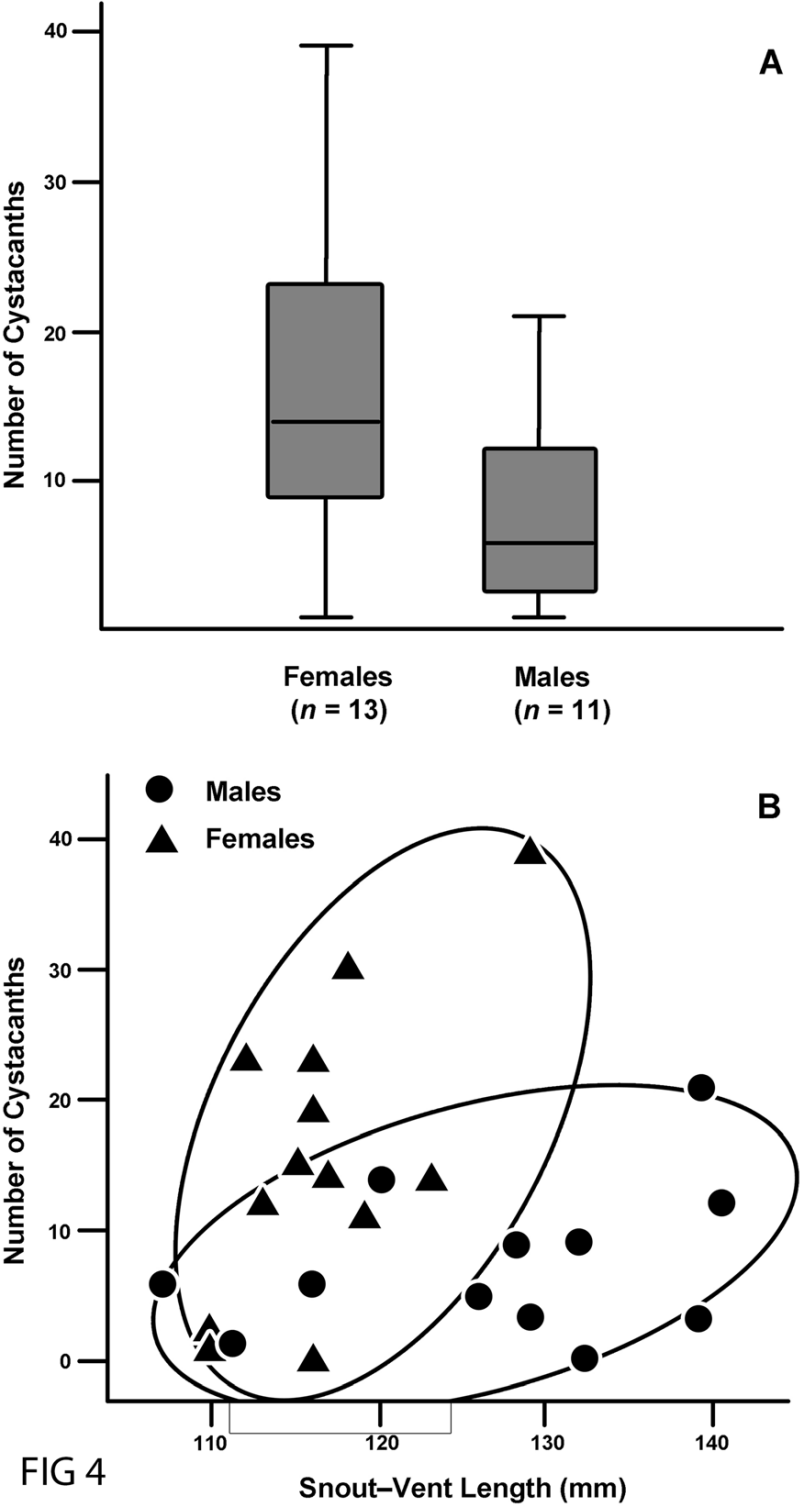
Ecological parameter of the infection with *Macracanthorhynchus ingens* in Brown Basilisks (**A**). Intensity of infection in adult females and males (**B**). Relationship between number of cystacanths and size of paratenic host.

The 33 Brown Basilisks from Florida contained 301 cystacanths of *M. ingens*. Most juveniles were uninfected (prevalence was 14%, *n* = 7); a single juvenile (SVL = 76 mm) contained two cystacanths. Among adult specimens, prevalence of this parasite in females (93%, *n* = 14) was similar to that in males (92%, *n* = 12). However, adult females contained more cystacanths than males (Welch’s *t*_14,12_ = 2.193, *P* = 0.040). In our samples, adult females contained 0–39 (15 ± 11, *n* = 14) cystacanths with an intensity of 16 ± 11 (*n* = 13), and males contained 0–21 (7 ± 6, *n* = 12) with an intensity of 8 ± 6 (*n* = 11) (Fig. 4A). In Brown Basilisks, cystacanths of *M. ingens* appear to have an aggregated population structure in both females (coefficient of dispersion = 8.3, *n* = 14) and males (coefficient of dispersion = 4.9).

Data for the females violated all three assumptions of linear regression analysis. Even if these violations are ignored, most females in our sample had similar snout-vent lengths (Fig. 4B), and, without additional specimens, we cannot determine if parasite load increases with size of female host. Males exhibited more variation in size, but data for males violated the assumption of linearity. Log-transformation of size did not remedy this problem. Nonetheless, if the assumption is ignored, we did not find a correlation between counts and SVL in males (*t*_12_ = 1.101, *P* = 0.297).

In our sample of Brown Basilisks, most cystacanths of *M. ingens* encysted in a retroperitoneal location. Although we did not note the specific location of each cystacanth collected, we found most within muscle fiber just outside of the posterior half of the coelom. In some heavily parasitized specimens, we found multiple cystacanths in muscle fibers ventral to and between transverse processes of the vertebrae and/or in musculature of the proximal thigh. Less often, cystacanths encysted between body wall musculature and the peritoneum of the anterior half of the body, in fat bodies, or in mesenteries.

## Discussion

The acanthocephalan *M. ingens* is a widespread parasite of North American carnivorous mammals (racoons, wolves, foxes, badgers, skunks, opossum, mink, and bears). However, two records, one in Nicaragua (Middle America) and Colombia (South America), suggested that *M. ingens* could be a species distributed across the Americas (Crum et al., 1978; Richardson, 2014; Hartnett et al., 2018). In the current study, three adult specimens were collected in two common racoons (*P. lotor*) in southeastern Mexico, representing the second record of this acanthocephalan in Mexico. In addition, we report new paratenic host records based on cystacanths recovered from two frog species from southeastern Mexico and Brown Basilisks in southern Florida. The cystacanths were initially identified as *M. ingens* based on their body shape, disposition of proboscis hooks (six rows with six hooks per row), and lemnisci extending to the posterior region with small nuclei (Figure 1A-D). However, we observed differences in the proboscis hooks sizes between the cystacanths from southeastern Mexico and southern Florida when compared to cystacanths described by Richardson (2005) (see Table 2). The morphological differences could be due to phenotypic plasticity (Roff, 2002; Miner et al., 2005), perhaps because each species of paratenic hosts (frogs and lizard) represents a different environment for the cystacanths.

The phylogenetic analyses inferred SSU and LSU datasets confirmed that the sequences of *M. ingens* from a cystacanth (hologenophore) from southern Florida is closely related with published sequences (AF001844, SSU and AY829088, LSU) of an adult specimen identified as *M. ingens* from southeastern Mexico (García-Varela et al., 2000; García-Varela & Nadler, 2005). However, both molecular markers showed slow substitution rates and the phylogenetic relationships of the species of *Macracanthorhychus* were weakly supported (see Fig. 2A–B). In contrast, the phylogenetic relationship inferred with *cox 1,* a molecular marker that has a fast rate of substitution, supported the monophyly of *Macracanthorhychus* (Fig. 3A). The *cox 1* sequences placed all the isolates of *M. ingens* generated in the current study in a single clade, together with an isolate identified as *M. ingens* available in GenBank (KT881244) from, Arkansas, USA. In addition, another subclade was formed by four isolates identified as *M. ingens* available in GenBank (KT881246-249) recovered from the Florida Ivory Millipede (*Chicobolus spinigerus*). The intraspecific genetic divergence among isolates from Mexico and the United States was very low, ranging from 0 to 2%. The level of intraspecific genetic variation found is similar to other archicanthocephalans. For example, the genetic divergence among four isolates of *Mediorhynchus gallinarum* (Bhalerao 1937), a parasite of birds from Asia, was 0.2% (Rodríguez et al., 2022); among 37 isolates of *Prosthenorchis elegans* (Diesing 1815), a parasite of New World primates and carnivores of South America, the intraspecific genetic divergence ranged from 0 to 1.6% (Falla et al., 2015). Finally, we found high genetic divergence (8 to 20%) among specimens identified as *M. ingens* from the Americas with one partial sequence identified as *M. ingens* (GenBank: ON197103) from a hedgehog from Iran. Based on the high genetic divergence and systematic position this sequence could not belong to *M. ingens*.

The haplotype network analysis of *cox 1* detected 10 distinct haplotypes obtained from 15 individual sequences. The haplotypes H1 and H2 were found in southeastern Mexico. The H1 was shared with two cystacanths recovered from

Vaillant´s Frog (*Lithobates vaillanti*) and Rio Grande Leopard Frog (*Lithobates berlandieri*) and from three adult specimens recovered in the gut of common raccoons. The haplotypes H3-H10 belong to cystacanths and an adult from the United Sates (Fig. 3B). The lack of shared haplotypes between Mexico and USA suggested that both populations are genetically isolated. The pattern of distribution of the haplotypes could be associate with the biology of the definitive host, as well the intermediate and paratenic hosts with more restricted capacity of dispersion.

Of the 15 frogs examined, two of seven Rio Grande Leopard Frogs, and three of eight Vaillant´s Frog were positive for cystacanths. Three cystacanths belonged to *M. ingens*, and two others belonged to genus *Oncicola* (see Fig. 3A). Both frog species showed low rate of infection (28% and 37% in Rio Grande Leopard Frog and Vaillant´s Frog, respectively). The presence of cystacanths of *M. ingens* and *Oncicola* sp., in the paratenic hosts resulted from the ingestion of arthropod the intermediate hosts. The current evidence suggested that the frogs are capable of harboring two species of archiacanthocephalans that complete their life cycle in different definite hosts. For example, the adult of *M. ingens* has been recorded only in raccoons and the adults of the genus *Oncicola* have been recorded in the Virginia Opossum (*Didelphis virginiana* Kerr) and White-Nosed Coati (*Nasua narica* Linnaeus) in southeastern Mexico (García-Prieto et al., 2010).

In addition, a total of 26 adults of Brown Basilisks (*Basiliscus vittatus*) from southern Florida, showed high rates of infection with *M. ingens* in 92% and 93% in males and females, respectively. In southern Florida, the Brown Basilisk is a successful and widespread invasive corytophanid lizard with breeding populations established as early as 1976 (Wilson & Porras, 1983; Krysko et al., 2006). This lizard inhabits riparian environments which are frequented by raccoons and other mammals such as foxes, skunks, opossum, coyotes and black bear which feed of brown basilisks, other lizards and frogs that serves as paratenic hosts of *M. ingens* (see references in Richardson, 2014). In addition, we found that, on average, female brown basilisks harbored twice as many cystacanths of *M. ingens* than males. The reason for this difference between the sexes is not known. However, relative to most lizards, this species is strongly dimorphic sexually (Rodda, 2020), and the difference in parasite load may reflect an ecological difference between males and females.

Finally, the participation of paratenic hosts in the life cycle of the acanthocephalans, represented an evolutionary innovation, and it has been conserved across the phylum (Kennedy, 2006; Near, 2002). When the cystacanth reaches a paratenic vertebrate host, it may partially evert and move into the body cavity, body wall, musculature, or some organ such as the liver where it encysts. At least in basilisks, we did not find cystacanths of *M*. *ingens* embedded in organ tissue, but observed cystacanths in all of these other locations with most being found in a retroperitoneal location. The paratenic host has played a principal role in the diversification of the acanthocephalans, because it facilitates transmission and serves as a trophic bridge between intermediate and definitive hosts (Kennedy, 2006).
